# Phonological awareness in Urdu language speaking children with down syndrome (DS)

**DOI:** 10.1371/journal.pone.0349773

**Published:** 2026-06-12

**Authors:** Maimoona Khalil, Nazia Mumtaz, Ghulam Saqulain, Farheen Naz Anis

**Affiliations:** 1 Department of Speech Language Pathology, Riphah International University, Lahore, Pakistan; 2 Department of ENT, Riphah International Hospital, Islamabad, Pakistan; Father Muller Charitable Institutions, INDIA

## Abstract

**Introduction:**

Phonological awareness (PA) is a vital part of literacy development. With dearth of evidence on phonological processing among Urdu-speaking children with down syndrome (DS), current study was conducted to determine the association between response time on rapid naming tasks and phonological awareness performance in children with Down syndrome.

**Methods:**

A quasi-experimental study that used cross-sectional design was carried out with 48 children with DS and who are Urdu speaking children (4–12 years) at three rehabilitation centers in Lahore, Pakistan. Rapid automatized naming and phoneme identification tasks were measured with the help of the Urdu Phonological Tele-Assessment (U-PASS) tool. Simple linear regression and partial correlation were analyzed.

**Results:**

The results revealed that the mean response time and phonological awareness had no significant relationship (R2 = 0.006, p = 0.587). The phoneme recognition (M = 9.42) at the beginning was much higher than the phoneme recognition (M = 5.42) at the end. There was moderate positive correlation existing between initial and final phoneme awareness (r = 0.423, p = 0.004) that did not depend on response time.

**Conclusion:**

Processing speed processing and phonological accuracy are two different cognitive constructs in children with DS. The clinicians are encouraged to emphasize the accuracy-based testing and adopt specific interventions addressing the end phoneme awareness.

## Introduction

Phonological awareness (PA) encompasses segmental and suprasegmental aspects of speech sound processing and it is a vital metalinguistic skill, which is important in the learning of literacies and effective communication [1]. Segmental PA involves perception and control of individual speech units which are individual phonemes and consonant-vowel pairs [2]. Besides segmental processing, suprasegmental processing properties of syllable awareness, stress pattern and prosodic boundaries also play a role in reading of both spoken and written language among children [3]. This phonological ability pyramid of increasing syllabic unit size, down to the decreasing phonemic unit size, plays a pivotal role in sound-symbol correspondence being inherent to the development of reading [4]. Processing efficiency as reflected in the processing speed of accessing and retrieving the phonological representations during rapid automatized naming (RAN) is an independent predictor of the literacy outcome [5]. Precision and speed in phonological processing are involved independently in reading competence, in which response time is an indicator of automaticity in retrieval of stored phonological representations [6].

The reason to explore the connection between RAN response time and phoneme identification lies in the two rival models. Shared-component hypothesis states that RAN and phonological awareness may be linked with a shared phonological representation system, which predicts that the quicker the RAN speed, the greater the accuracy of the phoneme identification should be [7]. The separability hypothesis, however, maintains that RAN exploits lexical retrieval speed [5], and phoneme identification involves explicit metalinguistic processing through controlled working memory and attention [4] implying that the two are variant constructs with different effects on literacy results [8].

This difference is especially important to children with Down syndrome, who are characterized by typical impairments in verbal short-term memory [9] and auditory processing, as well as fairly preserved visual processing and implicit learning [10]. This population may experience heightened dissociation of retrieval speed and explicit phonological analysis, because of the varied working memory requirements in these tasks [11]. Furthermore, phoneme-positioning is an additional cognitive burden: the initial phoneme recognition enjoys the support of acoustic salience and time precedence [12], whereas the terminal phoneme recognition demands the whole phonological image in working memory to isolate the last part [13].

In spite of this theoretical significance, a study has not analysed the relationship of RAN-phoneme identification among the children in the Urdu language and with Down syndrome. The current study has centred on studies primarily on English speaking typically developing populations [5] and has a gap on generalizability to languages with intellectual disabilities [14] and languages with different phonological fading such as Urdu that has retroflex consonants, difficult consonant clusters and Abjad system of writing [15].

Down syndrome (DS) is the most prevalent chromosomal disorder on a global scale with a prevalence rate of about 1 in 700–1000 live births [7]. DS occurs in 1:500–600 births in Pakistan, which is a high number based on which many educational and therapeutic services are needed for this population group [16]. Children who have DS show that they possess established gaps in phonological processing which have a bearing on their communicative ability and their ability to acquire literacy [17]. The research that has been conducted into the perception of PA in DS has predominately involved segmental speech perception, that is, phoneme identification and discrimination in studies [18]. However, there is scanty information regarding time dynamics of phonological processing among this population [13]. The trends of phonological development among Urdu-speaking children are not similar to English-speaking communities [15]. There is limited but growing research on Phonological awareness assessment and education resources for Urdu-speaking children with Down syndrome, hence there is not much research to accomplish diagnostics of the phonological ability in Urdu [19].

It is not until not long ago that the creation of linguistically-suited assessment devices such as the Urdu Phonological Tele-Assessment (U-PASS) instrument started to emerge [20]. Urdu Phonological Awareness Test was developed to assess phonological awareness in Urdu-speaking children [21]. Similarly, Articulation and Phonological Disorders Test (TAAPU) is designed to identify articulation and phonological difficulties [22].Measures of evaluation that also include processing time and error measures are poorly studied among DS populations [23]. Assessments involving technology have been useful in recording the fluctuations of phonological processing [8]. Nevertheless, there is a severe deficit of evidence on phonological processing among Urdu children with DS [14]. The segmental phoneme awareness activities, in particular, initial phoneme recognition, are very specific in their study, and the relationship between processing speed and phonological accuracy is not well known [24]. Information and communication technology application in phonological testing has not been applied systematically to Urdu speaking DS population [25]. Hence, some tools exist in Urdu language, specialized resources tailored to Down syndrome children remain scarce, highlighting need for culturally and linguistically appropriate interventions.

There is also a wide range in the phonological awareness results in the case of children with DS, yet the factors that lead to this variability are poorly understood [12]. Specifically, the linkage between the response time on rapid naming and ultimate phoneme identification, a more challenging phonological task as compared to initial phoneme awareness has never been examined among the children with the Urdu language and genetic disorder (DS) [26]. The assessment instruments must be validated to satisfy the requirement of this diverse linguistic population [27].

Therefore, the current research was carried out with the aim of establishing how response time in rapid naming activities correlates with phonological awareness in children with Down syndrome. The null hypothesis (H0) was that average response time on rapid naming tasks and phonological awareness scores in children with DS are statistically significantly unrelated to each other, and the alternative hypothesis (H1) was that these variables have a statistically significant relationship.

## Materials and methods

### Study design

Current cross-sectional quasi-experimental study was undertaken currently at three rehabilitation centers within Lahore, Pakistan in the form of Rising Sun Education and Welfare Center, Springfield Autism Center and Step Ahead Autism Center over five months, between 1st March to 31st July 2025.The design was chosen due to the lack of ethical possibility and practical viability in assigning participants to the group of diagnostic categories (Down syndrome verses typically developing) since children were presented with preexisting developmental disorders. The study involved naturally occurring correlations between response time on rapid tasks of naming and phonological awareness performance and not the impacts of testing interventions. This study design allowed a systematic exploration of phonological processing properties with the ability to inquire into the other significant covariates such as chronological age, cognitive level and language exposure history.

### Setting and participants

The data collection was carried out at three rehabilitations professionals’ inclusive centers in Lahore, Pakistan namely Rising Sun Education and Welfare Center, Springfield Autism Center and Step Ahead Autism Center. These centers were chosen specifically (with purposive selection) by distinguishing them by the definite programs with children having Down syndrome as well as having diagnostic records of the possibility to identify their status with respect to chromosomes. The duration of the study was increased to March 2025 up to July 2025, covering the procedure of hiring, administration of the assessment, and verification of the data.

### Determination of sample size

G*Power [28] analysis was used in order to establish the minimum required sample size in order to detect a medium effect size (f2 = 0.40) at alpha set at 0.05 and statistical power of 0.95. The test showed that 48 participants were required to be the minimum with 0.954 power to the planned regression analysis [29].

Inclusion criteria

1. Children with DS aged 3–12 years old with mild to moderate intelligence (IQ already calculated) were included as subjects in the study.
2. The participants were Monolingual Urdu speakers with their parents also utilizing Urdu language at their homes to guarantee consistent language exposure.
3. These criteria prevented the sample from being unrepresentative of the target population by ensuring that the sample was measuring phonological awareness and response time in children with DS who spoke Urdu.

Exclusion criteria

1. The study excluded children with comorbid conditions that could affect vocabulary such as hearing loss or autism spectrum disorder.
2. The exclusion of these other developmental and sensory challenges was essential to ensuring that the observed outcomes were linked specifically to DS and unrelated to them.

#### Participant flow.

**Fig 1:**
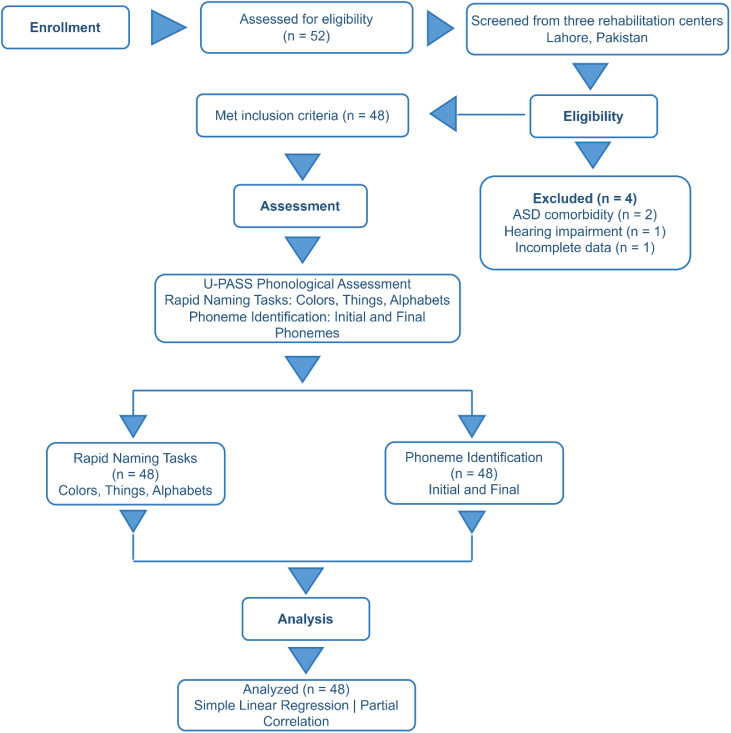
Participant’s flow diagram.

A total of 52 children with Down syndrome were first screened to be eligible at three rehabilitations in Lahore. Four children had been excluded; two because of comorbidity autism spectrum disorder, one because of severe hearing impairment and one because of incomplete assessment data. The ultimate sample size of the analysis was 48 participants that went through with all assessment elements (Fig 1).

### Assessment instrument: The urdu phonological tele-assessment tool (U-PASS)

Data collection involved two methods, including a simple demographic sheet and the Urdu Phonological Tele-Assessment Tool (U-PASS) [1,2]. The demographic sheet provided the data about age, sex, cognitive level (depending on already registered IQ scores), history of language exposure and affiliation to a center.

U-PASS is a linguistically and culturally sensitive pre-reading measure first designed by Bhalloo [14] and later validated to be used with children who are bilingual in Urdu-English [2,3]. This tool was created with a particular purpose to measure such phonological abilities of Urdu-speaking groups in which standardized assessment tools are not available or culturally unsuitable [30]. The U-PASS has three fundamental areas:

(a) **Phonological Awareness:** This area assesses the child to determine his/ her capacity of recognizing and controlling individual units of sound in spoken Urdu. It consists of two subtasks, the Primary one of which is (i) Initial Phoneme Identification, where a child is offered a target word and asked to determine the initial sound in the word using a set of response choices, and the Second task (ii) Final Phoneme Identification, where a child is forced to determine the last sound of a target word. The subtasks include 15 items apiece, which provides a maximum total of 15 on each subtask and a summative phonological awareness score of 30.
(b) **Rapid Automatized Naming (RAN):** This area assesses both the speed and efficiency of the lexical retrieval process by asking the child to name well-known visual objects as fast as possible. It consists of three subtasks (i) Rapid Naming of Colors where the child names a sequence of color patches arranged in a grid, (ii) Rapid Naming of Things (Objects) whereby the child names common objects appearing in a visual format as well as (iii) Rapid Naming of Alphabets whereby the child names Urdu alphabets presented sequentially. The response time of each subtask was measured in seconds with the help of a digital stopwatch, and the mean response time (ART) of all three subtasks was calculated as the main processing speed parameter.
(c) **Phonological Memory:** This area evaluates the capacity of the child to memorize and remember systems of sounds, which is central to the proper development of speech and reading. It is characterized by repetition of complicated non-word sequences which are composed of phonotactic rules of Urdu.

The phonological awareness domain (first and last phoneme recognition scores) in the current study was a dependent variable, whereas the average response time calculated in the three RAN subtasks was an independent variable. Phonological memory domain was added to the entire U-PASS battery, but not reflected in the main regression or partial correlation test, because it was not relevant to the focused study questions.

### Administration procedure for U-PASS

U-PASS was given to all the participants separately in a quiet room within the corresponding rehabilitation center. The researcher gave every item orally in Urdu, with visual stimulus (RAN-tasks: picture cards and phoneme identification tasks auditory prompts). Under the phonological awareness subtasks, the researcher read out each target word one at a time and gave three response choices and the child gave an answer loudly or by pointing at the choices. To attempt the RAN subtasks, the child was provided with a trial to practice pre-timing to ensure that he/she understood the task. The response time was taken between presenting the stimuli and when the child could name all the items in the grid. The scores were 1, 0 each depending on the right or wrong responses respectively. The results were picked to obtain composite scores on individual domains, which were then inputted into SPSS Version 24 to be analyzed further.

### Statistical analysis

Statistical package of Social Sciences (SPSS version 24) was used to perform the data analysis. The demographics and the task performance were included in descriptive statistics of means, standard deviations, and frequency distributions. The simple linear regression was used to determine the relationship between average response time (independent variable) and phonological awareness scores (dependent variable).

The theoretical justification of the selection of simple linear regression was presented in the Introduction. The shared-component hypothesis predicts that the response time of RAN should have a significant predictive relation on phonological awareness scores with significant R2 value and the separability hypothesis predicts a non-significant correlation with negligible R2 value. The analysis directly tested the relation between processing speed as a predictor of phonological accuracy (mean initial and final phoneme identification scores) by regressing phonological awareness scores on mean response time (mean RAN response time on color, object and alphabet naming tasks). The second partial correlation analysis involving initial and final phoneme identification scores including all three conditions of RAN response time variables assessed the existence of internal coherence of phonological awareness skills that do not depend on processing speed, i.e., whether children who do well in initial phoneme identification would also perform well in final phoneme identification. This two-step model (regression and then partial correlation) indicated the supportive evidence to the independence of processing speed and phonological accuracy in this population.

Partially correlated on the relationship between initial phoneme awareness and final phoneme awareness controlled possible confounding factors of age and level of cognitive in their analysis. The evaluation model fit was done using R-squared, F-statistics and analysis of residual distributions. The effects were computed and interpreted in line with conventional description. There were few missing data (<5%) that were addressed by list wise deletion.

### Ethical considerations

The Institutional Review Board of Riphah International University granted ethical approval to the study (Ref# REC/RCR & AHS/24/0641, dated 21 November, 2025). All the participants were provided with informed consent that was written after further explanations of the study procedures and the possible risks, benefits, and voluntary participation. In cases where child participants were of an age to provide verbal consent, verbal consent was obtained. Measures to ensure confidentiality such as coded identification, data security and access to identification data were also used. The participants were not penalized to withdraw at any point as it would not affect their continued clinical services..

## Results

Of the sample of 48 Urdu speaking down-syndrome children, the age distribution revealed that majority 18 (37.5%) fell in the age range of 8–9 Years (Table 1 with most being males 28 (58.3%) without significant differences in the proportion of both sexes across the age groups, with the exception of the youngest group.

**Table 1:** Demographic distribution of participants (N = 48).

Age Group	Total n (%)	Boys n (%)	Girls n (%)
4-5 years	**1 (2.1%)**	**1 (3.6%)**	**0 (0%)**
6-7 years	**13 (27.1%)**	**8 (28.6%)**	**5 (25.0%)**
8-9 years	**18 (37.5%)**	**10 (35.7%)**	**8 (40.0%)**
10-12 years	**16 (33.3%)**	**9 (32.1%)**	**7 (35.0%)**
Total	**48 (100%)**	**28 (58.3%)**	**20 (41.7%)**

### Primary outcome: Response time and phonological awareness

Simple linear regression was used to test the correlation between the average response time (ART) and the phonological awareness scores, which revealed no statistically significant relationship (R² = 0.006, F(1,46) = 0.299, p = 0.587). (Table 2).

**Table 2:** Regression Analysis – Response Time Predicting Phonological Awareness.

	R	R²	Adj. R²	SE Est.	F	p
Model	0.080	0.006	−0.015	5.670	0.299	0.587
Predictor	**B**	**SE**	**Β**	**T**	**p**	
Constant	17.678	5.264	—	3.358	0.002	
ART	−0.154	0.282	−0.080	−0.547	0.587	

Note: ART = Average Response Time; Dependent Variable = Phonological Awareness.

### Secondary outcomes: Phonological awareness subdomains

The descriptive statistics demonstrated that there were differences in performance on rapid naming and phoneme identification tasks (Table 3). Response time measures Rapid Naming of Alphabets (M = 19.35s, SD = 3.50), Rapid Naming of Things (M = 18.30s, SD = 5.20), and Rapid Naming of Colors (M = 17.60s, SD = 2.73) have highest mean.

**Table 3:** Descriptive Statistics of Key Variables (N = 48).

Variable	Mean	SD
Rapid Naming – Colors (seconds)	**17.60**	**2.73**
Rapid Naming – Things (seconds)	**18.30**	**5.20**
Rapid Naming – Alphabets (seconds)	**19.35**	**3.50**
Initial Phoneme Identification	**9.42**	**3.27**
Final Phoneme Identification	**5.42**	**3.48**

In the case of phonological awareness subdomains, the Initiate Phoneme Identification scored relatively higher (M = 9.42, SD = 3.27) than Final Phoneme Identification (M = 5.42, SD = 3.48), which means that children showed a better onset phonological awareness than coda phonological awareness.

### Effect modifiers: Partial correlation analysis

The partial correlation analysis was applied to assess the correlation between initial phoneme awareness and final phoneme awareness with the impact of all the three response-time variables being controlled. The initial phoneme performance was moderately positively correlated with final phoneme performance (r = 0.423, p = 0.004, df = 43), which implied that final phoneme performance was strongly predicted by initial phoneme performance regardless of processing speed (Table 4).

**Table 4:** Partial Correlation between Initial and Final Phoneme Awareness (Controlling for RT Variables).

Variables	Partial r	p-value	Df
Initial vs Final Phoneme	**0.423**	**0.004**	**43**

### Residual analysis

Poor fit to the model was verified through residual statistics. The phonological awareness values were predicted with limited values (13.46–15.65, SD = 0.45), as compared to the residuals, which were spreading significantly (range: −11.28 to 10.36, SD = 5.61). Standardized residuals were within reasonable limits (−1.989 to 1.828), but the large dispersion told us that a lot of unaccounted person-specific variation was not explained (Table 5).

**Table 5:** Residual Statistics.

Statistic	Minimum	Maximum	Mean	SD
Predicted Value	13.456	15.648	14.833	0.452
Residual	−11.280	10.365	0.000	5.610
Standardized Residual	−1.989	1.828	0.000	0.989

## Discussion

Study revealed no statistically significant association between average response time and phonological awareness (R² = 0.006, p = 0.587), indicating that processing speed and phonological accuracy are two independent cognitive constructs in Urdu-speaking children with Down syndrome. Second, the initial phoneme identification (M = 9.42) was showed to be much more effective in children than the final phoneme identification (M = 5.42), which is in line with the developmental patterns of phoneme recognition between onset and coda awareness. Third, moderate correlation between pre- and post-phoneme awareness remained significant when variables of response time were controlled (r = 0.423, p = 0.004) indicating that these phonological roles have underlying competency without reference to processing speed.

Variations in phonological awareness null the presence of any meaningful difference relating response time and phonological awareness, suggestive of the view that the faster the process, the more accurate the outcome in children with DS. This disassociation can be supported by the cognitive processes behind these constructs. Rapid automatized naming tasks are the ones that as a rule involve lexical retrievals activities and signify the effectiveness of retrieving stored phonological scripts [5]. Phonological awareness tasks, on the contrary, involve explicit metalinguistic analysis conscious manipulation of sound units that involves other cognitive resources [4]. These processes may be differentially affected by limitations of working memory that are typical of DS [11]. Children diagnosed with DS show certain losses of verbal short-term memory which might limit explicit phonological analysis but still afford retrieval automaticity [10].

The higher level on first compared to last phoneme tasks are consistent with inherent developmental patterns where onset awareness is preceded by coda awareness [12]. This tendency is an indication of significance of word-initial positions and acoustic salience. Among people with DS, the additional cognitive load of maintaining word-final position, and therefore, keeping the entire phonological code isolated over isolating the terminal position, can create specific problems due to reported limitations in the working memory [11]. An acoustic-phonetic nature of the Urdu language such as complex consonant clusters and retroflex can also make the final phoneme more difficult [13].

These results are congruent with the findings of Soccorso et al. [12], who have found that children with DS have phonological awareness skills with gross individual variation. This article builds on the overlaying the current research, in which Urdu-speaking groups are found to be the same, such that trends based on English-speaking groups in the rest of the language. In a comparable way, Onnivello et al. [10] have reported heterogeneous mental patterns among the children with DS, which has corroborated high inter-individual variance in our sample through all the measures.

The disconnection between the speed and accuracy of processing is similar to the results of Gray and Ehri [5], as they have stressed that fast automatized naming and phonological awareness are detachable foretellers of literacy achievement. Graziani et al. [8] showed that speed in naming can be increased by specific training in a sample of children with developmental difference, which implies that these skills are not fixed regardless of how well they are measured by accuracy metrics. This theoretical distinction is supported by our findings in the population of the DS in particular.

The phoneme position disparity performance also supports the finding of Ikhlaque and Yousaf [6] who discovered that Urdu-speaking children that are normally developing, also exhibit greater initial phoneme awareness. The current work projects these findings on children with DS and indicates that position effects in phonological awareness have similar developmental patterns irrespective of the status of intellectual disability. Schworer et al. [11] emphasized that working memory testing among young children with DS must take great caution in terms of task demands principle which equally applies to phonological awareness testing.

A study by Yasmin et al. [31] found certain trends in the deficit of receptive language and memory span in Pakistani children with developmental language disorders to have relevant information to interpret the cognitive profiles of the children in our sample of DS. The problems found in end phoneme awareness could be indicative of an identical underlying limitation of memory. Moreover, Iqbal and Nawaz [32] revealed that phonemic cueing issues have promise among the children with DS in Pakistani environment indicating the possibility of an intervention based on our results of position-specific challenges.

These results have significant implications to clinicians, educators, and assessment developers who deal with Urdu-speaking children with a DS. Speech-language pathologists are encouraged to understand that response time measures are not informative enough to reveal phonological competence; all-encompassing measures should be into accuracy-based measures in different positions of the phoneme. The highly expressed final phoneme awareness indicates that specific intervention to coda awareness should be made that could be based on visual aids and repetitive training to counteract the constraints of working memory [25]. Those teachers adopting literacy lessons must put more emphasis on initial phoneme awareness lessons before moving to more challenging final phoneme activities.

The usefulness of the U-PASS instrument in measurement of phonological skills in populations with developmental variances originating in the Urdu language was supported [2,3]. The use of technology based assessment tools provide good prospects of standardized assessment in linguistically diverse settings where no standardized assessment tools may be available or be culturally insensitive [24,27]. The developers of devices and tools should bear in mind that position-specific phoneme tasks with a descending level of difficulty can be used to reveal the entire spectrum of the phonological awareness skills.

The current research is one of the first examinations of phonological awareness among children with DS who speak Urdu, which is a major Literary gap. The quasi-experimental design offered ecological validity of an experiment based on real-life rehabilitation conditions instead of laboratory. The measurement validity was improved by the use of linguistically and culturally appropriate measure (U-PASS). The combination of various quick naming tasks (colors, things, alphabets) and initial and final phoneme awareness measures allowed studying the pattern of phonological processing in a more nuanced manner.

There are some shortcomings that deserve attention. The quasi-experimental design was cross-sectional, which did not allow the use of random allocation, thereby allowing the risk of selection bias. The sample size of the study (N = 48) might have had low statistical strength to measure smaller effects and limited its generalizability. Three rehabilitation centers in Lahore were used to draw the sample, which might not be representative of other geographic areas or other service settings. The other possible confounding factors such as educational exposure, home reading environment and individual intervention history were not controlled in a systematic manner. Lack of a usual developing comparison group did not allow direct comparisons of development.

Further studies need to use more and more geographically focused samples so that it would be more representative of the Pakistani and other Urdu-speaking communities. Longitudinal designs would shed light on developmental patterns of phonological awareness in children with DS and establish the best intervention periods. Research that included multilingual speakers of the Urdu-English language would discuss the multilingualism in which these children grow [26]. Research into particular intervention techniques that aim to enhance final phoneme awareness, control of comparison conditions, would put evidence-based practices into place in this group [33]. The neural processes of the identified dissociation between the processing speed and the phonological accuracy may be clarified by the means of integration of neuroimaging methodologies.

## Conclusion

Processing speed and phonological accuracy are two different cognitive constructs among the kids of Urdu language who have Down syndrome. Children exhibited superior initial phoneme awareness than the final phoneme identification, which is in accordance with the normal developmental development. Such findings demonstrate the validity of holistic phonological evaluation by accuracy and not by speed measures of processing. The significant lack of final phoneme awareness demonstrates that speech-language pathologists have to apply specific interventions to the population and emphasizes the importance of the culture and language-specific assessment tools within various communities.

## Supporting information

S1 Data Data file. (XLSX)
